# Sub-regional disparities in the use of antenatal care service in Mauritania: findings from nationally representative demographic and health surveys (2011–2015)

**DOI:** 10.1186/s12889-021-11836-z

**Published:** 2021-10-09

**Authors:** Gebretsadik Shibre, Betregiorgis Zegeye, Bright Opoku Ahinkorah, Dina Idriss-Wheeler, Mpho Keetile, Sanni Yaya

**Affiliations:** 1 Department of Reproductive Health and Health Services Management, School of Public Health, Addis Ababa University, Addis Ababa, Ethiopia; 2 HaSET Maternal and Child Health Research Program, Shewarobit Field Office, Shewarobit, Ethiopia; 3 School of Public Health, Faculty of Health, University of Technology Sydney, Ultimo, Australia; 4 Faculty of Health Sciences, University of Ottawa, Ottawa, Ontario Canada; 5 Population Studies and Demography, University of Botswana, Gaborone, Botswana; 6 School of International Development and Global Studies, Faculty of Social Sciences, University of Ottawa, 120 University Private, Ottawa, ON K1N 6N5 Canada; 7 The George Institute for Global Health, Imperial College London, London, UK

**Keywords:** Antenatal care, Inequality, Mauritania, DHS, Global health

## Abstract

**Background:**

Skilled antenatal care (ANC) has been identified as a proven intervention to reducing maternal deaths. Despite improvements in maternal health outcomes globally, some countries are signaling increased disparities in ANC services among disadvantaged sub-groups. Mauritania is one of sub-Saharan countries in Africa with a high maternal mortality ratio. Little is known about the inequalities in the country’s antenatal care services. This study examined both the magnitude and change from 2011 to 2015 in socioeconomic and geographic-related disparities in the utilization of at least four antenatal care visits in Mauritania.

**Methods:**

Using the World Health Organization’s Health Equity Assessment Toolkit (HEAT) software, data from the 2011 and 2015 Mauritania Multiple Indicator Cluster Surveys (MICS) were analyzed. The inequality analysis consisted of disaggregated rates of antenatal care utilization using four equity stratifiers (economic status, education, residence, and region) and four summary measures (Difference, Population attributable risk, Ratio and Population attributable fraction). A 95% Uncertainty Interval was constructed around point estimates to measure statistical significance.

**Results:**

Substantial absolute and relative socioeconomic and geographic related disparities in attending four or more ANC visits (ANC4+ utilization) were observed favoring women who were richest/rich (PAR = 19.5, 95% UI; 16.53, 22.43), educated (PAF = 7.3 95% UI; 3.34, 11.26), urban residents (D = 19, 95% UI; 14.50, 23.51) and those living in regions such as Nouakchott (R = 2.1, 95% UI; 1.59, 2.56). While education-related disparities decreased, wealth-driven and regional disparities remained constant over the 4 years of the study period. Urban-rural inequalities were constant except with the PAR measure, which showed an increasing pattern.

**Conclusion:**

A disproportionately lower ANC4+ utilization was observed among women who were poor, uneducated, living in rural areas and regions such as Guidimagha. As a result, policymakers need to design interventions that will enable disadvantaged subpopulations to benefit from ANC4+ utilization to meet the Sustainable Development Goal (SDG) of reducing the maternal mortality ratio (MMR) to 140/100, 000 live births by 2030.

## Background

Globally, more than 800 women still die each day from pregnancy and childbirth-related complications [1]. Although the global maternal mortality rate (MMR) decreased from 451, 000 to 295, 000 between 2000 and 2017 [2], more than two-thirds (68%) of these deaths or nearly 200,000 maternal deaths took place in sub-Saharan Africa [2]. In 2017, an estimated 1100 women died during childbirth in Mauritania with a high MMR) (766 deaths per 100,000 live births) compared to sub-Saharan Africa (542 deaths per 100,000 live births) [2, 3].

Skilled antenatal care (ANC) is one of the vital services provided to pregnant women to enhance likelihood of safe pregnancy outcomes for the mother and fetus [4, 5]. Pregnant women have the opportunity to use services such as nutrition, health checks, services that detect pregnancy risks, counselling and support services for women and their families, and skilled birth attendants which often lead to positive pregnancy outcomes or lower maternofetal deaths [6–8]. Numerous studies have shown the positive effect of ANC on the birth weight of a child [9–12], early detection of foetal abnormalities including the diagnosis of growth retardation [13, 14] and decrease in maternal and neonatal morbidity and deaths [15–17]. In addition, ANC coverage is a good measure for maternal health care service access and utilization during pregnancy [18–21].

Several studies in Africa have identified socioeconomic factors such as household wealth status, maternal education, subnational region, and place of residence as factors that influence ANC services [22–28]. Literature in Mauritania shows that maternal health services, including ANC, can be affected by factors such as maternal education, household economic status, place of residence, distance to facility, parity and previous adverse pregnancy outcomes [29, 30].

There is evidence of disparities within and across countries in maternal health services, particularly ANC four coverage (i.e., the percentage of women aged 15–49 with a live birth in a given time period that received ANC four or more times during their pregnancy) [31–34]. Recent studies in low-and-middle-income countries show that ANC four coverage varied by at least 25 percentage points between the least and most educated as well as the poorest and richest women, while ANC one coverage varied by at least 10 percentage points between women in the richest and poorest subgroup in half of the countries [34]. We anticipate similar findings in Mauritania.

Available evidence in Mauritania highlighted disparities in maternal and child health services from a one-time survey completed in 2012 [29, 30]. In an effort to update the knowledge and investigate the change overtime, we used the recommended and rigorous inequalities techniques available through WHO’s Health Equity Assessment Toolkit (HEAT) that houses ANC4 + data on Mauritania from 2011 and 2015 [35]. The HEAT toolkit helps to investigate inequalities in an indicator, such as ANC4+ utilization, using a wide range of inequality measures. This type of in-depth examination in inequality remains an important first step before running the traditional multivariable regression analysis. This study aimed to answer two main questions. First, what are the extent of socioeconomic, urban-rural and subnational disparities in ANC4 + in Mauritania? Second, what are the socioeconomic, urban-rural and sub-national disparity changes in ANC4+ utilization from 2011 to 2015?

## Method

### Data source

This study used data from the 2011 and 2015 Mauritania Multiple Indicator Cluster Survey (MICS). MICS started in the mid-90s by UNICEF to provide data on tracking progress toward the Millennium Development Goals (MDGs) and Sustainable Development Goals (SDGs), particularly those related to maternal-child health and education [36]. The Mauritania MICS 2011 is part of MICS4 that interviewed 12,754 women ages 15–49 from 10,116 households and gathered data on 9278 children under five [37]. The Mauritania MICS 2015 also forms part of MICS5 by which successful interviews were conducted among 14,342 women aged 15–49, 4691 men aged 15–49 from 11,765 households. Additionally, 10,663 questionnaires were administered to gather data on children under five [38]. The female questionnaire included different topics such as maternal health, maternal health care services such as antenatal care, and knowledge of HIV/AIDS. Finally, our sample included the 3569 and 4149 women, in 2011 and 2015 respectively, aged 15 to 49 years who received any ANC in 2 years preceding the surveys for their most recent birth.

### Selection of variables

The outcome variable of interest in this study was ANC4+ utilization which uses data from the number of antenatal care visits made by a pregnant woman (i.e., count form 1, 2, 3 … n) [39]. We defined ANC4+ utilization in this study as the percentage of women with a birth in the 2 years preceding the survey who received ANC four or more times during the pregnancy. Women who received the service from medical doctors, nurses, midwives are said to have received skilled ANC. Moreover, the questions about ANC were asked during the most recent live birth. The data used in this study were collected before the updated WHO new recommendation of at least eight ANC contacts, therefore, we used the original WHO benchmark of at least four antenatal care visits for ANC utilization by pregnant women in this study [40, 41].

### Equity stratifiers

To measure inequality of ANC4+ utilization, we used the equality stratifiers of economic status, educational status (no education, primary and secondary/higher education), place of residence (rural, urban) and subnational region. As described in previous studies using DHS and MICS data, wealth index (classified as poorest, poor, middle, rich and richest) was calculated using Principal component Analysis (PCA) of household assets and other characteristics to approximate economic status [42]. There were 13 subnational regions in Mauritania, namely, Hodh charghy, Hodh Gharby, Assaba, Gorgol, Brakna, Trarza, Adrar, Dakhlett Nouadibou, Tagant, Guidimagha, Tirs-ezemour, Nouakchott Inchiri and Nouakchott.

### Inequality measures

We measured the disparities of ANC4+ utilization using two steps. First, ANC4+ utilization was disaggregated using the above described equity stratifiers of economic status, education status, place of residence and subnational region. Second, we examined disparities in ANC4+ utilization using inequality measures of Difference, Population Attributable Risk (PAR), Population Attributable Fraction (PAF) and Ratio. While PAR and PAF are complex measures, Difference and Ratio are simple measures. Ratio and PAF measured relative inequalities, while Difference and PAR measured absolute inequalities.

Based on the recommendation of previous studies highlighting the use of relative and absolute as well as single and complex measures in a single study, our selection of summary measures was in compliance with and used all recommended measures [35, 43]. Using various types of summary measures may lead to differing conclusions; ignoring these findings can confuse or possibly negatively impact the decision-making process [35, 43] . Unlike simple measures, complex measures are preferred because they account for the size of categories in a sub-population, which is important for over-time analysis [35]. The advantages of simple measures are that they are easier to explain, interpret and understand by multiple stakeholders. Consequently, having simple and complex as well as relative and absolute measures in an inequality study provides a more accurate and realistic perspective to decision and policy making.

### Statistical analysis

We used the 2019 WHO’s HEAT software (version 3.1) to analyze the data [44]. The HEAT software technical notes [44] and the WHO handbook on health inequality monitoring [43] outline detailed calculations regarding inequality measures. In summary, Difference (D) and Ratio (R) were calculated for each of the equity measures to render absolute and relative simple measures, respectively. If D rendered a value of zero (0) or R a value of 1, inequality did NOT exist [35, 43, 44]. For economic status, D was the richest group minus poorest group while R was the richest group divided by the poorest group. In education, the secondary/higher educated group minus uneducated group rendered D while R was the division of the former by the latter. We subtracted rural from urban for place of residence D and divided urban by rural for R. The difference for Subnational region included the region with highest ANC4+ utilization coverage minus region with lowest ANC4+ utilization, and Ratio (R) was calculated by dividing the two subgroups.

A value of zero (0) for PAR or PAF indicated inequality did NOT exist and the greater absolute value of both these complex measures indicated higher inequality. The PAR (the difference between the ANC4+ utilization estimate for the reference group and the national average) used the most disadvantaged subgroup as the reference group. For the ordered categorical dimensions, we used the richest subset for economic status, and the uneducated subset as the reference group for education, comparing it to the national average. Both the binary dimension of the place of residence (rural) and the non-ordered categorical dimension of the subnational region with the highest estimate (Dakhlett Nouadibou for 2011 and Nouakchott for 2015) were used as the reference. To calculate PAF, we divided PAR by the national average (μ) and multiplied by 100 [31, 32].

We computed 95% Uncertainty Intervals (UI) around point estimates of each measure for each survey year to: (i) examine whether ANC4+ utilization shows statistically significant disparities across the sub-groups of each equity stratifer, and (ii) determine whether or not the inequality changed over time. For inequality measures other than Ratio and PAF, the lower and upper bounds of the UI must not include zero to interpret that inequality exists. For Ratio and PAF, the interval should not include one. We assessed the changes of inequality for each summary measure by referring to the UIs for the different survey years; if the UIs did not overlap, inequality changed over time. The analysis took into account the complex nature of the MICS dataset including unequal probability of selection. For instance, the HEAT software applied “weighting” to rectify the problem of differing selection probability during the multi-stage selection of samples.

### Ethical consideration

The WHO Health Equity Monitor database draws on Demographic and Health Survey (DHS) and the Multiple Indicator Cluster Survey (MICS) data from 95 countries [35]. Data analysis for this study is from publicly available MICS data set. Since the ethical clearance was approved by the institution that commissioned, funded and managed the overall UNICEF MICS program, further ethical approval was not required. Informed consent from the participants before the survey was obtained during the course of the survey. United Nations Children’s Fund, National Office of Statistics (Mauritania) and relevant ethical review board of Mauritania were responsible to ensure and comply with appropriate ethical research protocols.

## Results

Overall a total of 3569 and 4149 women in 2 years preceding the surveys participated in the 2011 and 2015 surveys, respectively. These women constituted those who had at least one live birth the 2 years preceding the survey. Of them, 4383 (56.8%) were rural residents, and 1622 (21%) were from the poorest (quintile) subgroups. More than one-fourth (26%) and 2071 (26.8%) of the respondents had no formal education and attended primary school, respectively.

Table 1 shows the socioeconomic, rural-urban and regional disparities in ANC4+ utilization across subpopulations in Mauritania from 2011 to 2015, with higher coverage among the advantaged subgroups such as quintile 5 (richest), educated, urban residents and women from certain regions such as Nouakchott. ANC4+ utilization significantly varied across wealth quintiles with greater utilization among quintiles 5 and quintile 4 compared to quintiles 1 and 2.

**Table 1:** Percentage of women who used ANC4+ in 2011 and 2015 by socioeconomic and geographic characteristics

Dimension of Inequality	Subgroup	2011		2015	
		Estimate (95% UI)	women	Estimate (95% UI)	women
Economic status	Quintile 1 (poorest)	34.68 (30.39, 39.24)	748	47.22 (41.87, 52.65)	874
	Quintile 2	39.93 (36.11, 43.87)	728	53.32 (48.50, 58.08)	870
	Quintile 3	51.95 (47.58, 56.29)	694	63.22 (59.00, 67.25)	824
	Quintile 4	53.57 (49.41, 57.69)	754	71.79 (68.04, 75.25)	833
	Quintile 5 (richest)	63.91 (58.93, 68.61)	644	82.43 (77.85, 86.24)	746
Education	No education	38.47 (34.55, 42.54)	882	57.33 (52.46, 62.07)	1131
	Primary school	49.49 (45.87, 53.12)	1256	54.29 (49.32, 59.18)	815
	Secondary school +	62.20 (57.82, 66.38)	618	63.65 (60.49, 66.70)	1463
Place of residence	Rural	43.17 (40.26, 46.13)	2101	54.40 (50.86, 57.89)	2282
	Urban	55.83 (52.27, 59.34)	1468	73.41 (70.47, 76.15)	1867
Subnational region	01 Hodh charghy	44.02 (37.57, 50.67)	401	52.98 (44.21, 61.58)	499
	02 Hodh Gharby	26.93 (20.01, 35.19)	274	49.79 (42.79, 56.81)	409
	03 Assaba	52.16 (44.67, 59.55)	379	57.10 (51.20, 62.81)	476
	04 Gorgol	41.19 (34.88, 47.80)	335	58.25 (50.59, 65.53)	505
	05 Brakna	49.71 (41.24, 58.20)	322	68.59 (62.07, 74.45)	425
	06 Trarza	63.44 (57.77, 68.76)	350	72.38 (65.47, 78.36)	297
	07 Adrar	29.51 (23.11, 36.83)	79	48.34 (34.17, 62.78)	19
	08 Dakhlett Nouadibou	64.38 (57.68, 70.57)	107	73.59 (65.71, 80.21)	135
	09 Tagant	27.14 (20.23, 35.36)	98	49.27 (41.39, 57.20)	22
	10 Guidimagha	33.50 (26.68, 41.08)	254	38.68 (30.26, 47.82)	326
	11 Tirs-ezemour	49.97 (42.32, 57.63)	70	58.07 (45.85, 69.38)	19
	12 Nouakchott [12 Inchiri^a^]	57.83 (52.45, 63.03)	895	70.49 (54.57, 82.61)	5
	[13 Nouakchott^a^]	NA	NA	80.34 (76.14, 83.95)	1005

^a^ Rearranged regions for 2015 surveys. *NA* Not applicable for the 2011 survey. *UI* Uncertainty Interval

The coverage increased from 2011 to 2015 across all wealth quintiles. However, a higher increment among quintile 5 (18.5 percentage points [pp]) and quintile 4 (18.2 pp) were observed compared to quintiles 1 (12.5 pp) and 2 (13.4 pp).

The utilization of ANC was also higher among secondary school/higher subpopulations compared to uneducated and primary schools. ANC4+ utilization significantly increased among the uneducated (18.9%) from 2011 to 2015, whereas among primary and secondary school subgroups, it increased by 4.8 pp. and 1.5 pp., respectively.

Similarly, a noticeable gap was seen between urban and rural settings in both survey years; higher ANC4+ utilization was observed among urban residents both in 2011 and 2015, and, the change between the subpopulation were dissimilar. Utilization increased by 17.6 pp. for urban residents and, to lesser extent, 11.2 pp. for rural residents over the 4 year period.

Similarly, ANC4+ utilization varied across regions from 2011 to 2015. In the Guidimagha region, the coverage increased only by 5.2 pp., whereas in the Brakna region, there was an 18.9 pp. increase (Table 1).

### Magnitude of inequality

Table 2 shows significant socioeconomic, residence and subnational regional disparities in ANC4+ utilization in Mauritania from 2011 to 2015, favoring women with higher socioeconomic status, urban residents and regions such as Nouakchott. Significant absolute and relative wealth-driven disparities in ANC4+ utilization have been observed from 2011 to 2015 using simple (D, R) and complex (PAR, PAF) measures, favoring quintile 5 (richest) compared to quintile 1 (poorest) and quintile 2 (poorer).

**Table 2:** Socioeconomic and area-based disparities in ANC4+ utilization by the different inequality measures in Mauritania in 2011 and 2015

Dimension		2011	2015
	Measure	Estimate (95% UI)	Estimate (95% UI)
Economic status	D	29.22 (22.66, 35.78)	35.20 (28.39, 42.02)
	PAF	32.09 (25.71, 38.47)	30.94 (26.25, 35.63)
	PAR	15.53 (12.44, 18.61)	19.48 (16.53, 22.43)
	R	1.84 (1.56, 2.11)	1.74 (1.52, 1.96)
Education	D	23.72 (17.87, 29.58)	6.31 (0.60, 12.03)
	PAF	27.41 (21.89, 32.94)	7.30 (3.34, 11.26)
	PAR	13.38 (10.68, 16.08)	4.33 (1.98, 6.68)
	R	1.61 (1.41, 1.81)	1.11 (1.00, 1.21)
Residence	D	12.66 (8.06, 17.25)	19.01 (14.50, 23.51)
	PAF	15.40 (12.54, 18.26)	16.60 (14.46, 18.74)
	PAR	7.45 (6.07, 8.83)	10.45 (9.10, 11.80)
	R	1.29 (1.17, 1.41)	1.34 (1.24, 1.45)
Region	D	37.45 (27.46, 47.43)	41.66 (32.00, 51.31)
	PAF	33.07 (22.46, 43.67)	27.61 (19.49, 35.73)
	PAR	16.00 (10.87, 21.13)	17.38 (12.27, 22.49)
	R	2.39 (1.67, 3.10)	2.07 (1.59, 2.56)

*D* Difference. *R* Ratio. *PAF* Population Attributable Fraction. *PAR* Population Attributable Risk

Substantial disparities were observed across all the dimensions from 2011 to 2015. The Difference measure in 2011 (29.22, 95% UI; 22.66, 35.78) and 2015 (35.20, 95% UI; 28.39, 42.02) indicate significant absolute economic-related disparities favoring the advantaged subpopulation (richest). Similarly, the PAF measure in 2011 (32.09, 95% UI; 25.71, 38.47) and 2015 (30.94, 95% UI; 26.25, 35.63) show substantial relative economic-related disparities in ANC4+ utilization disfavoring the disadvantaged subpopulation (poorest) overtime.

The current study also shows substantial absolute and relative education-related disparities in ANC4+ both in 2011 and 2015 using all four measures (D, PAF, PAR, R) favoring the advantaged subpopulation (secondary schools and higher) (Table 2). The pattern of education disparities decreased over the last 4 years in ANC4+ utilization. Although PAR dropped from 13.38 (95% UI; 10.68, 16.08) in 2011 to 4.33 (95% UI; 1.98, 6.68) in 2015, salient absolute education-related disparities in ANC4+ utilization existed favoring women who attended secondary school or higher compared to those who were uneducated. Similarly, the Ratio measure (1.61, 95% UI; 1.41, 1.81 and 1.11, 95% UI; 1.00, 1.21) in 2011 and 2015, respectively, indicated significant relative education-related disparities in ANC4+ utilization favoring subgroups with secondary or higher education.

Significant absolute and relative rural-urban inequality in ANC4+ utilization were observed in 2011 and 2015 using both simple (D, R) and complex (PAF, PAR) measures favoring women living in urban areas. PAR not only showed a significant difference between urban and rural; it also showed a significant increase between 2011 and 2015 from 7.45 (95% UI; 6.07, 8.83) to 10.45 (95% UI; 9.10, 11.80), also favoring urban regions. Measures of Difference, PAF and Ratio, revealed constant rural-urban disparity pattern in ANC4+ utilization in both years, but no significant change overtime from 2011 to 2015. For example, the PAF measure (15.40, 95% UI; 12.54, 18.26 and 16.60, 95% UI; 14.46, 18.74) in 2011 and 2015 surveys, respectively, indicate substantial relative urban-rural disparities in ANC4+ utilization with higher service uptake among urban residents compared to their rural counterparts, but without over time change as the confidence intervals of the two-time period did overlap.

Substantial absolute and relative subnational regional inequality were observed both in 2011 and 2015 using simple (D, R) and complex (PAF, PAR) measures. The pattern of regional disparities was constant with no change over time from 2011 to 2015 survey points. For example, the Difference measure - 37.45 (95% UI; 27.46, 47.43) in 2011 and 41.66 (95% UI; 32.00, 51.31) in 2015 - demonstrated substantial absolute regional disparities in ANC4+ utilization favoring regions such as Dakhlett Nouadibou, but no change overtime. Similarly, the PAF measure (33.07, 95% UI; 22.46, 43.67 and 27.61, 95% UI; 19.49, 35.73) in 2011 and 2015 surveys, respectively, suggest significant relative regional inequality in ANC4+ utilization with no change over time (see Table 2 for more details).

## Discussion

This study highlights the magnitude and change from 2011 to 2015 in socioeconomic and rural-urban inequalities as well as regional variations in antenatal care four service utilization. Substantial economic, education, urban-rural and regional disparities exist, favoring pregnant women who are from the richest quintile, educated, residing in urban settings and regions such as Nouakchott. We explored the disparities in ANC4+ utilization in Mauritania using HEAT, one of the most useful health equity assessments tools developed, recommended, and used internationally [43]. Evaluation of disparities regarding a health care indicator (i.e., ANC4+ utilization) using HEAT provides policymakers and researchers with valuable information on the prevailing status of that disparity within the country. The tool helps identify gaps among subgroups for interventions, policies, and programs regarding the indicator of interest across various dimensions of inequality (i.e., maternal education, transportation, health coverage), contributing to national priority-setting in health [35]. Furthermore, it allows investigation of the change of inequality overtime to create context specific strategies for areas where inequities have not changed to better serve the different segments of a population, particularly the most disadvantaged groups. Key measures (PAF and PAR) were selected due to their powerful ability to “show the potential for improvement in setting average that could be achieved if all the population subgroups had the same level of the indicator as the most advantaged (reference) group” [ [35], p.4].

Mauritania, categorized as a low-and-middle income country (LMIC), has unacceptably high maternal deaths [2, 3], therefore, antenatal care is a vital intervention to saving maternal and neonatal lives [4, 5]. Evidence from other LMICS [45–49] highlighted the need for equity in maternal health services within a country and demonstrated that researching or working towards increasing the national coverage alone may aggravate within country disparities, overlooking those most in need, and stalling the movement towards achieving key maternal child health SDG targets [50]. Differences in socioeconomic status and geographic location disadvantage a significant number of pregnant women in LMICs who are not receiving ANC services, yet this is easily masked by national coverage numbers that show improvements overtime [45–49].

Consistent with previous studies [22, 24, 26, 28, 51], we noticed disproportionately higher ANC 4+ utilization among richest and rich women compared to poorest and poor women. In 2015, ANC4+ utilization among the richest women was higher by, on average, 35 percentage points compared to the poorest women. A possible reason for this disparity, as suggested by other studies, could be poverty - known to be a barrier to healthcare utilization in Sub-Saharan Africa (SSA) - because poor women are unable to afford the medical and non-medical costs associated with utilizing services such as ANC [52, 53]. This can limit the number of ANC visits, reduce early ANC initiation or prevent pregnant women from attending ANC entirely [26, 51]. Although some African countries offer free or subsidized maternal health services, pregnant women still have to pay for some direct medical costs (i.e., laboratory testing) and non-medical costs (i.e., transport to appointments) which become hindrances to using ANC services [54, 55].

Interestingly, a recent study by Ravit et al. (2020) looked at the impact of the obstetrical risk insurance (ORI) scheme on maternal healthcare utilization in Mauritania. ORI is a pre-payment scheme for pregnant women that is not based on risk pooling and regular payments, but rather focuses on an attempt to improve access to healthcare for pregnant women by lowering direct payments and increasing likelihood of using ANC (Ravit et al., 2020). Previous work had shown ORI availability at the district level had increased skilled ANC and delivery rates in health centres [56]. According to Ravit et al. (2020), the availability of ORI in the district levels is not enough; women need to be enrolled to improve use of health services during pregnancy. Barriers for not participating in ORI were aligned with some of the findings in this study that prevent many women from accessing ANC – access to ORI in their district, not deemed important/poor quality of health services, cost too high, distance/transportation and poor information [57].

Better ANC4+ utilization was found among women who attended secondary school and above, compared to women who had no formal education and attended primary school. This is similar to previous studies in SSA [22, 24, 28]. Studies have explained that education provides autonomy and empowers women with the capacity to make informed and responsible decisions about their health [48, 58]. Educated women also have the advantage of a better understanding of health information given to them by health professionals regarding the importance of the continuum of maternal care [48]. In addition, educated women are also empowered to become more financially independent through employment and are thus able to afford the cost of ANC services [59].

Pro-urban disparities in service utilization of ANC4+ was evident in both survey years. Findings imply that if both absolute and relative urban-rural inequalities were avoided, the 2015 national ANC four utilization could potentially increase by approximately 10 percentage points and 17%, respectively. Similar findings were observed in previous studies [46, 60, 61]. This could be due to better quality ANC services provided as well as the short distance to the healthcare facilities in urban regions [46, 49]. In rural areas, there is limited distribution of health services, poor educational and employment status of residents as well as inadequate access to media, all of which are possible barriers to ANC utilization [45]. Compared to rural residents, studies have shown that urban residents generally have better knowledge regarding obstetric danger signs due to access to the media or health professionals, which increases their odds of attending ANC [62]. Similarly, in addition to associated costs, walking a long distance is a challenge for pregnant women and may discourage them from using ANC services [47]. This negative effect of long distance on the utilization of ANC and the continuum of maternal healthcare services has been documented in other studies [47, 49].

Another significant finding from the current study is the regional disparities in ANC uptake. ANC4+ utilization among women living in Nouakchott region were higher by 42 percentage points as compared to women living in the Guidimagha region. The finding also confirmed that the utilization among women in the Nouakchott region were 2 times higher than women residing in the Guidimagha region. Previous studies also confirmed regional variations in ANC coverage [63, 64]. Possible reasons could be differences in accessibility to ANC services, road and transportation access, affordable healthcare capacity, accessibility to health facilities, skilled health personnel and quality of care in the health care facilities [65, 66].

### Strength and limitations of the study

The strength of this study is the use of WHO’s HEAT to examine inequality in ANC4+ utilization using simple and complex as well as absolute and relative measures. This approach provides various perspectives using different measures to assist policymakers in understanding the degree of disparity that exists among sub-groups, particularly outlining where a disproportionately higher or lower concentration of services exist. Additionally, there is comparability of findings across published papers using the same techniques, sources, and data through HEAT. However, the study has limitations. Methods did not include the analysis of explanatory factors for ANC utilization disparities. Future work could include studies that investigate factors that may explain the inequalities across different equity stratifiers.

## Conclusions

The study examined not only the magnitude of disparities in ANC4+ utilization, but also assessed over time dynamics of disparities in Mauritania. As anticipated, and similar to findings in other regions of SSA, there was disproportionately lower service uptake of ANC4+ among women who were poor, uneducated, living in rural areas and regions such as Guidimagha. There is the need for policymakers to design interventions that will enable disadvantaged subpopulations to benefit from antenatal care services to meet the Sustainable Development Goals (SDG) so all countries should aim to reduce the maternal mortality ratio (MMR) to less than 140/100, 000 live births by 2030 [67]. Further studies are essential to investigate factors that lead to inequities in ANC utilization in emerging nations like Mauritania to make informed decisions that mitigate the existing or emerging issues using limited resources.

## Data Availability

The datasets generated and/or analyzed during the current study are available in Health Equity Assessment Toolkit: Previous versions (who.int).

## Abbreviations

ANC: Antenatal Care
D: Difference
MICS: Multiple Indicator Cluster Survey
EA: Enumeration Area
HEAT: Health Equity Assessment Toolkit
ICF: Inner City Fund
PAF: Population Attributable Fraction
PAR: Population Attributable Risk
PCA: Principal Component Analysis
R: Ratio
SDG: Sustainable Development Goal
UI: Uncertainty Interval
WHO: World Health Organization
